# *Zingiber striolatum* phytochemicals ameliorated hyperglycemia symptoms by modulating gut microbial communities in mice with type 2 diabetes mellitus

**DOI:** 10.3389/fnut.2025.1537932

**Published:** 2025-01-22

**Authors:** Tingting Liu, Xiaodong Ge, Lu Song, Huanhuan Wu, Xue Qian, Bowen Jia, Chao Zhao, Yi Zhuang

**Affiliations:** ^1^Department of Clinical Pharmacy, The Second People’s Hospital of Yancheng, Yancheng, China; ^2^College of Marine and Bioengineering, Yancheng Institute of Technology, Yancheng, China; ^3^Department of Clinical Pharmacy, The First People’s Hospital of Yancheng, Yancheng, China; ^4^Department of Clinical Pharmacy, Suqian Hospital Affiliated to Xuzhou Medical University, Suqian, China; ^5^College of Marine Sciences, Fujian Agriculture and Forestry University, Fuzhou, China

**Keywords:** type 2 diabetes mellitus, *Zingiber striolatum*, gut microbiota, hyperglycemia, ethanol extract

## Abstract

**Introduction:**

Prolonged hyperglycemia caused by type 2 diabetes mellitus (T2DM) can lead to liver injury and disrupt the community of the gut microbiota that pose significant risks to human health. As a food rich in a variety of active ingredients, *Zingiber striolatum* (*Z. striolatum*) exhibits hypoglycemic and hypolipidemic effects. However, the regulatory influence of *Z. striolatum* ethanol extract (ZSE) on the gut microbiota of T2DM mice or its potential relationship with T2DM pathology remains unexplored.

**Methods:**

After a one-week acclimation period, 12 mice were randomly selected as the normal group. The remaining 48 mice were employed T2DM model, and then randomly assigned to four groups: the model group, a low-dose ZSE group (ZSE-L, 100 mg/kg/day), a high-dose ZSE group (ZSE-H, 300 mg/kg/day), and a positive control group treated with metformin hydrochloride (MET, 100 mg/kg/day).

**Results:**

After a 4-week intervention, the results revealed that ZSE significantly ameliorated fasting blood glucose (FBG), area under the curve of oral glucose tolerance test (AUC of OGTT) and glycated serum protein (GSP) in T2DM mice. Moreover, the high-dose (ZSE-H) treatment increased the relative abundance of beneficial bacteria such as *Faecalibaculum*, while reducing harmful bacteria such as *Bilophila*, thereby alleviating insulin resistance. Additionally, ZSE-H demonstrated superior efficacy over low-dose (ZSE-L) in improving FBG, AUC of OGTT, and other hypoglycemic parameters. Predictive analysis of the correlation between gut microbiota and hypoglycemic parameters identified *Dubosiella*, *Bacillus*, and *Mailhella* as potential microbial biomarkers for further investigation into the pathogenesis of T2DM.

**Conclusion:**

ZSE plays a pivotal role in mitigating hyperglycemia in T2DM mice through the modulation of intestinal microbiota communities.

## Graphical abstract

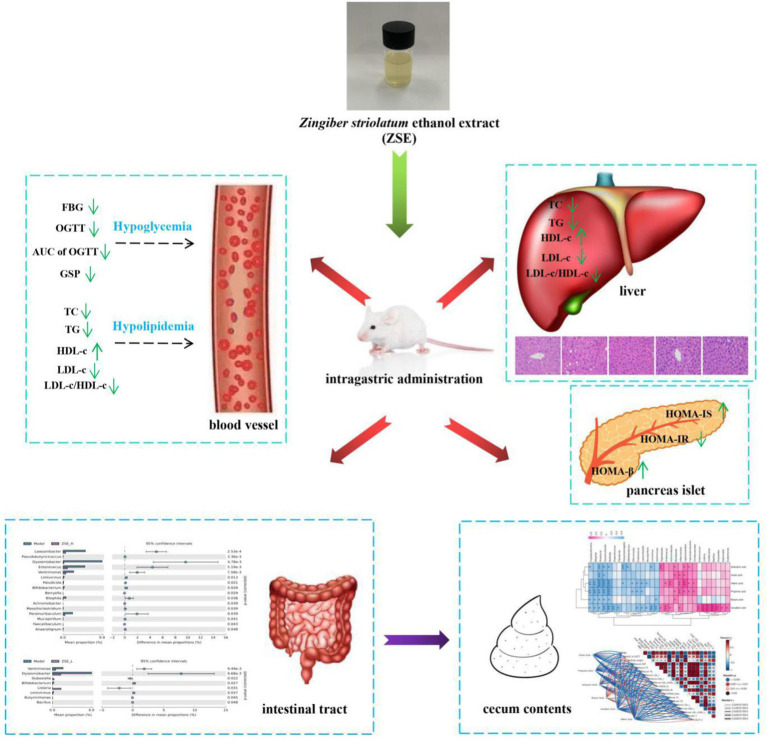

## Highlights

- ZSE ameliorated the symptoms of hyperglycaemia in T2DM mice.
- ZSE improved the pancreatic islet dysfunction in T2DM mice.
- ZSE regulated the proportion of intestinal microbiota associated with T2DM.

## 1 Introduction

As an endocrine and metabolic disorder, diabetes primarily arises from insufficient insulin secretion or abnormal pancreatic islet function, leading to persistently elevated blood glucose level (1, 2). In recent years, with rapid socioeconomic growth and significant improvements in living standards, the incidence of diabetes among Chinese residents has been rising steadily, posing a substantial threat to both quality of life and life expectancy. This increasing prevalence is gradually evolving into a critical global health issue with serious implications (3). Data from the International Diabetes Federation (IDF) reveals a concerning trend: between 2019 and 2021, the global number of adults (aged 20–79) with diabetes increased from approximately 463 million to 537 million, a growth rate of 15.98%, while in China, the diabetic population rose from 116.4 million to 140.9 million, reflecting a 21.05% increase, ranking China first in the world (4, 5). Particularly troubling is the fact that more than 90% of these individuals have been diagnosed with type 2 diabetes mellitus (T2DM), a condition in which long-term hyperglycemia progressively damages the liver, intestines, and cardiovascular system, resulting in a series of irreversible complications that severely threaten human health and quality of life (6). Therefore, enhancing the management and research of hyperglycemia in T2DM has become a pressing need to improve public health and promote sustainable societal development.

In recent years, the connection between intestinal microbiota and human health has gained increasing attention. Numerous studies have demonstrated that the diversity of intestinal microbial communities and fluctuations in the relative abundance of bacterial genera are intricately linked to the onset and progression of T2DM, underscoring the pivotal role of gut microbiota in T2DM regulation (7, 8). Our previous research has revealed a significant correlation between glucose metabolism disturbances and alterations in intestinal microbial communities (9, 10). Specifically, at the phylum level, an elevated Firmicutes-to-Bacteroidetes (Firmicutes/Bacteroidetes) ratio disrupts glucose metabolism and exacerbates insulin resistance. At the genus level, an increase in beneficial intestinal bacteria effectively boosts the production of short-chain fatty acids (SCFAs) in the intestines of T2DM mice, thereby alleviating hyperglycemia. Moreover, changes in the relative abundance of *Lactobacillus*, *Parabacteroides*, *Lachnoclostridium*, and *Desulfovibrio* are closely associated with key biochemical indicators in T2DM mice, including total cholesterol (TC), triglycerides (TG), low-density lipoprotein cholesterol (LDL-c), high-density lipoprotein cholesterol (HDL-c). Chen et al. (11) demonstrated that *Gymnema sylvestre* extract regulates the relative abundance of *Clostridium*, *Lactobacillus*, *Bacteroides*, and enhances acetic and butyric acid content, leading to improved hyperglycemic symptoms in T2DM rats. Similarly, based on the gut-liver axis theory, Cui et al. (12) found that *Hericium erinaceus* polysaccharides increased the relative abundance of *Dubosiella*, *Akkermansia*, and *Lactobacillus*, with *Dubosiella* and *Lactobacillus* showing a negative correlation with liver injury. Additionally, impaired intestinal barrier function due to microbial dysbiosis significantly raises the risk of T2DM progression and contributes to multi-organ injury (13). Jiang et al. (14) found that petroleum ether extract of *Schisandra sphenanthera* improved intestinal barrier integrity and permeability by modulating the abundance of *Lactobacillus*, *Prevotella*, and other bacteria, ultimately alleviating liver and pancreatic injury in T2DM rats. Consequently, dysregulation of intestinal microbiota, marked by decreased beneficial bacteria and increased harmful bacteria, has emerged as a promising target for the treatment of T2DM and its related complications.

*Zingiber striolatum* (*Z. striolatum*), a perennial herb from the ginger family, has gained widespread attention in recent years due to its rich nutrient profile and unique biological properties. It has become a focal point for researchers in food, agriculture, and nutrition (15, 16). *Z. striolatum* contains bioactive compounds such as polysaccharides, volatile oils, flavonoids, and natural pigments, offering multiple health benefits, including hypoglycemic, hypolipidemic, anticoagulant, antiviral, anti-infective, and anti-aging effects (17, 18). However, previous studies have primarily focused on the extraction processes and physiological functions of *Zingiber striolatum* polysaccharides. For instance, Jiang et al. isolated and purified *Z. striolatum* pure polysaccharide-1 (ZSPP-1), which modulated the Firmicutes/Bacteroidetes ratio and regulated the abundance of *Akkermansia*, *Lactobacillus*, and *Bacteroides* in the gut of obese mice, thereby restoring microbial balance disrupted by a high-fat diet. Despite these findings, no studies have yet explored the regulatory effects of *Z. striolatum* ethanol extract (ZSE) on the gut microbiota of T2DM mice or its potential relationship with T2DM pathology. In this study, a T2DM mouse model was successfully established by a high-sugar and high-fat (HSHF) diet combined with intraperitoneal streptozotocin injections to evaluate the effects of ZSE on hyperglycemia symptoms and to further investigate its impact on intestinal microbial communities. This research provides a novel perspective on the potential therapeutic applications of ZSE in managing T2DM and lays a solid foundation for future studies on *Z. striolatum* as a natural antidiabetic resource.

## 2 Materials and methods

### 2.1 Preparation and analysis of ZSE

Based on previously established methods, the extraction procedure for ZSE was optimized (19, 20). Specifically, dried *Z. striolatum* was pulverized and sieved through a 40-mesh screen. The resulting powder was immersed in a 70% ethanol solution (1:45, w/v) and subjected to reflux extraction at 70°C for 4 h. After the extraction, the supernatant was collected, and the remaining precipitate underwent a second extraction with an equivalent volume of 70% ethanol under the same conditions. The combined supernatants from both extractions were filtered and subjected to vacuum distillation, followed by freeze-drying to obtain ZSE for subsequent animal experiments. To identify the primary chemical constituents of ZSE, UPLC-QTOF-MS/MS (Thermo Fisher Scientific, United States) was employed for analysis.

### 2.2 Animal experiments

Sixty healthy male ICR mice (SPF, 4 weeks old, 20 ± 4 g) were obtained from the Comparative Medical Center of Yangzhou University. The mice were housed in a controlled temperature and humidity barrier system (23 ± 2°C) with a 12-h light/dark cycle to simulate day and night. Food and water were provided ad libitum to maintain normal physiological rhythms. After a one-week acclimation period, 12 mice were randomly selected as the normal group and continued on a basal maintenance diet. The remaining 48 mice were placed on a HSHF diet for intervention. After 4 weeks, the 48 mice received intraperitoneal injections of streptozotocin (35 mg/kg) dissolved in 0.1 M citrate buffer (pH 4.5) three times per week, while the normal group received the same dose of 0.1 M citrate buffer. The T2DM model was considered successfully established if fasting blood glucose (FBG) level was ≥11.1 mM, measured 48 h after the final injection. The T2DM mice were then randomly assigned to four groups: the model group (*n* = 12), a low-dose ZSE group (ZSE-L, 100 mg/kg/day, *n* = 12), a high-dose ZSE group (ZSE-H, 300 mg/kg/day, *n* = 12), and a positive control group treated with metformin hydrochloride (MET, 100 mg/kg/day, *n* = 12). This design allowed for the comparison of therapeutic effects between different ZSE dosages and the conventional drug metformin hydrochloride. The normal and model groups received 100 mg/kg/day of deionized water. All mice had free access to food and water and received daily oral gavage for 4 weeks. Throughout the intervention, the health status of the mice was closely monitored and recorded, with body weight and FBG levels measured weekly.

Following 4 weeks of continuous intragastric administration, an oral glucose tolerance test (OGTT) was conducted on T2DM mice to comprehensively assess their glucose metabolism capacity. Blood samples were collected from the tail vein using a glucose meter to determine FBG level (G_0h_). Subsequently, all mice were administered a glucose solution (2 g/kg body weight) by gavage to simulate postprandial glucose increase following the intake of sugary foods. Additional blood samples were collected at 0.5, 1, and 2 h post-gavage (G_0.5h_, G_1h_, and G_2h_), and blood glucose levels were measured at these time points.

To quantify the glucose metabolism trends observed during the OGTT, the area under the curve of OGTT (AUC of OGTT) was calculated, reflecting the degree of glucose accumulation following glucose administration. The formula used for calculation was: AUC of OGTT = 0.25 × (G_0h_ + G_0.5h_) + 0.25 × (G_0.5h_ + G_1h_) + 0.5 × (G_1h_ + G_2h_). The night prior to dissection, all mice were deprived of food. The following day, the mice were anesthetized using a combination of sutazine (55 mg/kg body weight) and cyrazine hydrochloride (5 mg/kg body weight). Blood samples were collected via orbital extraction, and the mice were sacrificed by cervical dislocation. The blood samples were allowed to rest for 2 h before being centrifuged (1,000 g, 10 min) to obtain serum for subsequent biochemical analysis to evaluate the physiological status of the mice and the therapeutic effects of ZSE. Liver and cecal content samples were collected; a portion of the liver tissue was immediately fixed in 4% paraformaldehyde for histopathological examination. The remaining liver tissue and cecal content samples were frozen in liquid nitrogen and stored in an ultra-low temperature freezer for future analysis.

### 2.3 Homeostasis model assessment insulin-related indicators

Fasting insulin (FINs) level in the serum samples prepared in section 2.2 were measured using an ELISA kit (Wuhan Chundu Biotechnology Co., Ltd., China), following the manufacturer’s instructions. The indices related to pancreatic islet function were calculated by the following formulas: HOMA-β (pancreatic islet β-cell function index) = 20 × FINs/(FBG − 3.5); HOMA-IR (insulin resistance index) = FINs × FBG/22.5; and HOMA-IS (insulin sensitivity index) = 1/(FINs × FBG) (21).

### 2.4 Analysis of serum and liver biochemical indicators

In addition to the serum samples, liver tissue was homogenized with 0.9% normal saline (1:9, w/v), and the supernatant was collected after homogenization. Various biochemical indicators in the serum and liver supernatant were measured according to the protocols provided by Nanjing Jiancheng Bioengineering Institute (China). These indicators included TC, TG, LDL-c, HDL-c, and glycated serum protein (GSP), which are essential for evaluating glucose and lipid metabolism as well as liver function.

### 2.5 Histopathological analysis

Liver tissue samples were fixed using the paraffin embedding technique, after which 4 μm-thick sections were cut using a microtome (Nikon, Japan). The liver sections were subsequently stained with hematoxylin and eosin, and the histopathological characteristics of the liver cells were observed under a microscope (Nikon, Japan). Histopathological scoring of liver sections was performed according to the criteria proposed by Kleiner et al. (22), which assess several factors, including steatosis, inflammation, and fibrosis. These indicators were used to determine a final score that evaluates the pathological state and extent of liver injury.

### 2.6 qPCR and immunohistochemistry

The 0.1 g liver tissue sample was quickly ground into a fine powder and transferred to a 1.5 mL centrifuge tube. Total RNA was extracted using TRIzol reagent (Invitrogen, United States). cDNA synthesis was performed with the RevertAid First Strand cDNA Synthesis Kit (Takara, Japan). Quantitative PCR (qPCR) was carried out using SYBR^®^ Premix Ex Taq^™^ II (Takara, Japan) on an ABI 7500 fluorescence quantitative PCR instrument (Applied Biosystems, United States). β-actin was used as the internal reference gene, and specific primers were designed for AKT-1 and GLUT-2 (Table 1). The qPCR reaction conditions were as follows: initial denaturation at 95°C for 5 min, followed by 40 cycles of 95°C for 15 s and 60°C for 60 s, with a final extension at 60°C for 5 min. Relative mRNA transcription levels were calculated using the $2^{-\Delta \Delta C_{T}}$ method.

**Table 1:** List of all primers used for qPCR.

Primer name	Forward primer (5′–3′)	Reverse primer (5′–3′)
β-actin	TGTCCACCTTCCAGCAGATGT	AGCTCATAACAGTCCGCCTAGA
AKT-1	ACTCATTCCAGACCCACGAC	CCGGTACACCACGTTCTTCT
GLUT-2	TACGGCAATGGCTTTATC	CCTCCTGCAACTTCTCAAT

For immunohistochemistry, after dewaxing the liver tissue sections, they were incubated for 15 min with 3% hydrogen peroxide to inhibit endogenous peroxidase activity and reduce non-specific staining. The sections were then rinsed with deionized water, followed by antigen retrieval to enhance antibody binding specificity. After antigen retrieval, the sections were washed with PBS to remove any residual solution and impurities. Rabbit polyclonal antibodies against AKT-1 (Cat. No. 10176-2-AP) and GLUT-2 (Cat. No. 20436-1-AP) from Proteintech (China), diluted 1:100, were applied to the sections. The sections were incubated overnight at 4°C to ensure optimal binding of the primary antibody to the target antigen. The following day, the sections were incubated in a HRP-conjugated goat anti-rabbit IgG solution for 30 min, allowing the secondary antibody to specifically bind to the primary antibody attached to the target antigen. The sections were then dehydrated with a graded ethanol series to remove moisture, followed by counterstaining with hematoxylin to enhance tissue structure and cellular morphology. Finally, the sections were sealed with neutral resin to preserve them from environmental impact. The sections were analyzed using an image analysis system of Nikon Eclipse TE2000-U (Nikon, Japan). Semi-quantitative histochemistry scoring (H-score) was applied to evaluate the staining intensity of each section using the following formula: H-score = (strongly positive percentage × 3) + (moderately positive percentage × 2) + (weakly positive percentage × 1) (23).

### 2.7 Analysis of cecal microbiota

Metagenomic DNA was extracted from the cecal content using the QIAamp-DNA Stool Mini Kit (Qiagen, Hilden, Germany). PCR products were isolated from a 2% agarose gel, purified using the AxyPrep DNA Gel Extraction Kit, and quantitatively analyzed using a fluorescence photometer. The purified and quantified PCR products were subsequently sequenced on the Illumina NovaSeq PE250 high-throughput platform. The raw sequencing data underwent processes such as splicing and filtering to generate valid data. Operational Taxonomic Units (OTUs) cluster analysis was then performed on the valid data. Based on the OTU clustering results, species classification analysis was conducted to assess the composition, abundance, and diversity of the microbiota. Sequencing results were analyzed as described by Huang et al. (20). To further investigate the potential relationships between hyperglycemic parameters and intestinal microbiota at the genus level, Spearman’s correlation analysis was performed using R software (version 4.1.0). Heat maps were generated to visually present the correlation results. For parameters exhibiting particularly strong correlations (|*r*| ≥ 0.6), a visual correlation network was constructed using Cytoscape software (version 3.9.0).

### 2.8 Determination of SCFAs in the cecum

Standard solutions of acetic acid, propionic acid, isobutyric acid, butyric acid, isovaleric acid, and valeric acid were prepared by accurately measuring the compounds and placing them into 10 mL volumetric flasks. Anhydrous ether was added to each flask to create individual standard stock solutions. These solutions were further diluted by a double-gradient method with anhydrous ether to prepare six standard solutions at varying concentrations, which were then analyzed by gas chromatography. A standard curve for each SCFAs was generated by plotting the concentration of the standard solutions against their corresponding gas chromatography peak areas. Cecal content samples (100 mg) were thoroughly mixed with 1 mL of deionized water to ensure even dispersion in the solvent. Phosphoric acid was added, and the mixture was vortexed vigorously for 2 min. SCFAs were extracted using anhydrous ether and filtered through a 0.22 μm nylon filter before being analyzed by gas chromatography (Shimadzu, Kyoto, Japan). The content of SCFAs was determined by referring to our previous work (10). Chromatographic conditions were as follows: HP-INNOWAX capillary column chromatography (30 m × 0.25 mm × 0.25 μm), with high-purity nitrogen as the carrier gas at a flow rate of 1 mL/min. The injection volume was 1 μL, with an injector temperature of 260°C, a detector temperature of 260°C, and a split ratio of 10:1. The temperature program started at 100°C (held for 1 min), followed by an increase to 200°C at a rate of 5°C/min, where it was maintained for 2 min.

### 2.9 Statistical analysis

All data were collected in triplicate and expressed as mean ± standard deviation (SD). Statistical significance was determined using one-way analysis of variance (ANOVA) with Tukey’s post-hoc test, conducted via SPSS 16.0. A *p*-value of less than 0.05 was considered statistically significant.

## 3 Results

### 3.1 Composition of ZSE

In this study, 15.40 g of ZSE was extracted from 100 g of *Z. striolatum*. Using UPLC-QTOF-MS/MS, a total of 2,388 compounds were identified (ESI^+^: 1270, ESI^−^: 1118). The major bioactive components were identified as flavonoids (49), including nobiletin, chrysin, luteolin, and hyperoside; quinolines and derivatives (31), such as quinate, dibucaine, and 4-hydroxyquinoline; indoles and derivatives (66), including lactic acid, indolelactic acid, and tryptophan; prenol lipids (110), such as β-eudesmol, linalool, camphor, and isoalantolactone; isoflavonoids (18), including wedelolactone, formononetin, genistein, and mefenamic acid; and phenols (61), such as sinapyl alcohol, 4-bromophenol, phenylephrine, and 4-hexylresorcinol (Supplementary Table S1).

### 3.2 Effects of ZSE on body weight, FBG, OGTT, and serum GSP

**Figure 1:**
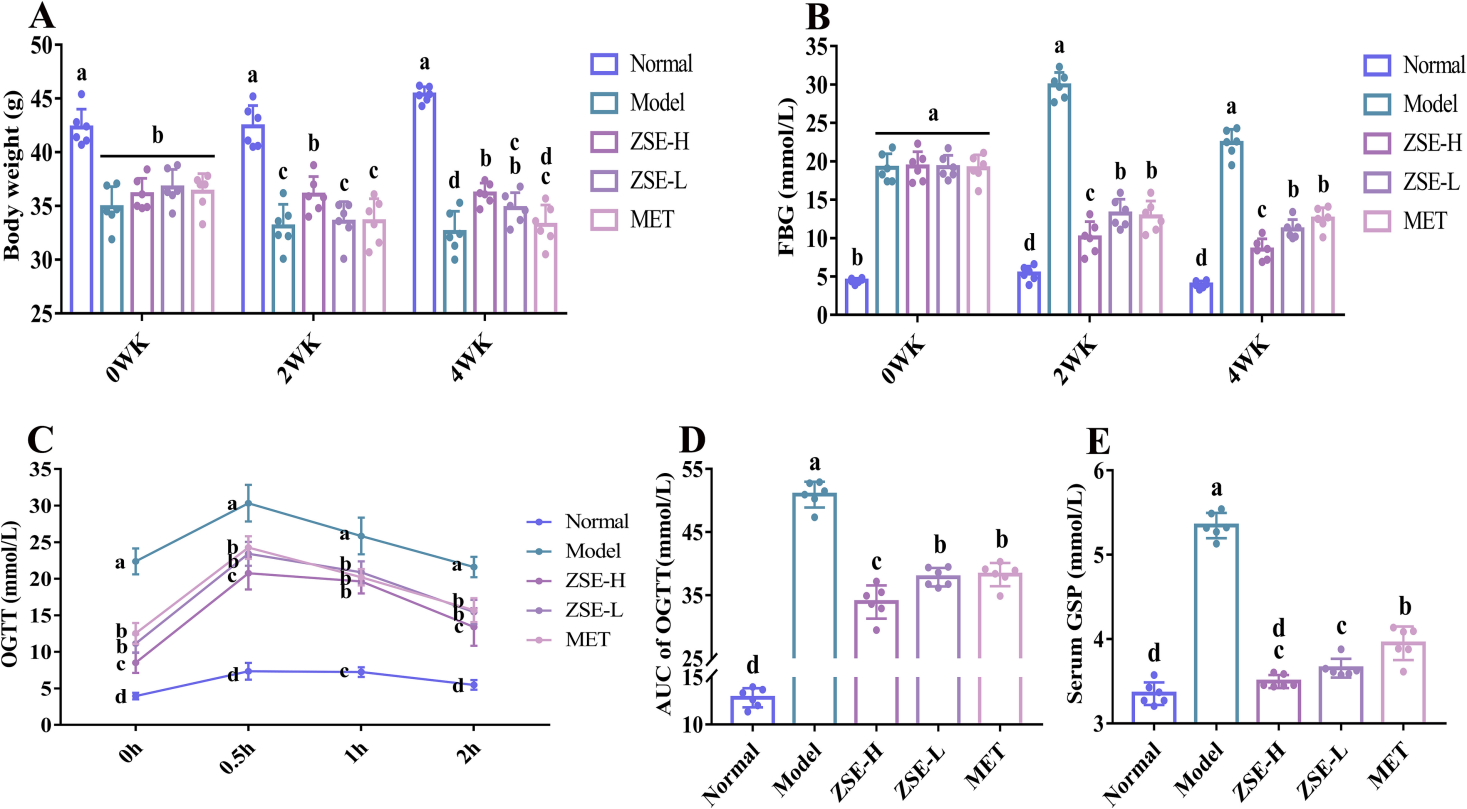
Effects of ZSE treatment during the experimental period. **(A)** Body weight, **(B)** FBG, **(C)** OGTT, **(D)** AUC of OGTT, and **(E)** serum GSP. ZSE refers to *Zingiber striolatum* ethanol extract, and T2DM indicates type 2 diabetes mellitus. WK stands for week. Different superscript letters represent statistically significant differences between groups (*p* < 0.05).

At week 0, the body weight of all groups, except the normal group, was significantly reduced compared to the normal group (*p* < 0.05) (Figure 1A). After 2 weeks of ZSE intervention, the body weight in the model, ZSE-H, ZSE-L, and MET groups remained significantly lower than that of the normal group (*p* < 0.05). Additionally, compared to the ZSE-H group, the body weight in the model, ZSE-L, and MET groups was significantly lower (*p* < 0.05). By week 4, the body weight in all other groups continued to be significantly lower than the normal group (*p* < 0.05), but the ZSE-H and ZSE-L groups showed significantly higher body weights than the model group (*p* < 0.05). The improvement trend in FBG level in T2DM mice treated with ZSE over 0–4 weeks is clearly illustrated (Figure 1B). At week 0, the FBG levels in the model, ZSE-H, ZSE-L, and MET groups were significantly higher than those in the normal group (*p* < 0.05), with no significant differences between these four groups (*p* > 0.05). After 2 weeks, FBG levels in the ZSE-H, ZSE-L, and MET groups were significantly lower compared to the model group (*p* < 0.05), with the ZSE-H group showing significantly lower FBG level than the ZSE-L and MET groups (*p* < 0.05), although still higher than the normal group (*p* < 0.05). After 4 weeks of ZSE intervention, the ZSE-H group exhibited significantly lower FBG level than both the ZSE-L and MET groups (*p* < 0.05).

The oral glucose tolerance capacity of T2DM mice was illustrated (Figure 1C). After 0.5 h of intragastric administration of a 2 g/kg body weight glucose solution, blood glucose level in all mice peaked and then gradually declined. The OGTT curves of the ZSE-H, ZSE-L, and MET groups were situated between those of the normal and model groups. Specifically, at 0 h, the blood glucose level in the normal group was significantly lower than in the other groups (*p* < 0.05), whereas the model group exhibited significantly higher levels compared to the other groups (*p* < 0.05). After 0.5 h of glucose administration, the blood glucose level in all groups reached their highest point, with the ZSE-H, ZSE-L, and MET groups showing significantly lower levels than the model group (*p* < 0.05). At 1 and 2 h, blood glucose level in all groups gradually decreased, with the ZSE-H, ZSE-L, and MET groups remaining significantly higher than the normal group (*p* < 0.05), but significantly lower than the model group (*p* < 0.05). Notably, the blood glucose level in the ZSE-H group was significantly lower than the ZSE-L and MET groups at 0, 0.5, and 2 h (*p* < 0.05). To directly quantify and assess glucose metabolism in the T2DM mice, the AUC was calculated to evaluate blood glucose fluctuations during the OGTT (Figure 1D). The AUC of OGTT for the ZSE-H, ZSE-L, and MET groups was significantly lower than that of the model group (*p* < 0.05). Additionally, the AUC of OGTT for the ZSE-H group was significantly lower than that of the ZSE-L and MET groups (*p* < 0.05). In Figure 1E, serum GSP level was significantly reduced in all treatment groups compared to the model group (*p* < 0.05). Interestingly, the serum GSP level in the ZSE-H group was comparable to those of the normal group, with no significant difference observed (*p* > 0.05).

### 3.3 Effects of ZSE on HOMA index and serum biochemical indicators of T2DM mice

**Figure 2:**
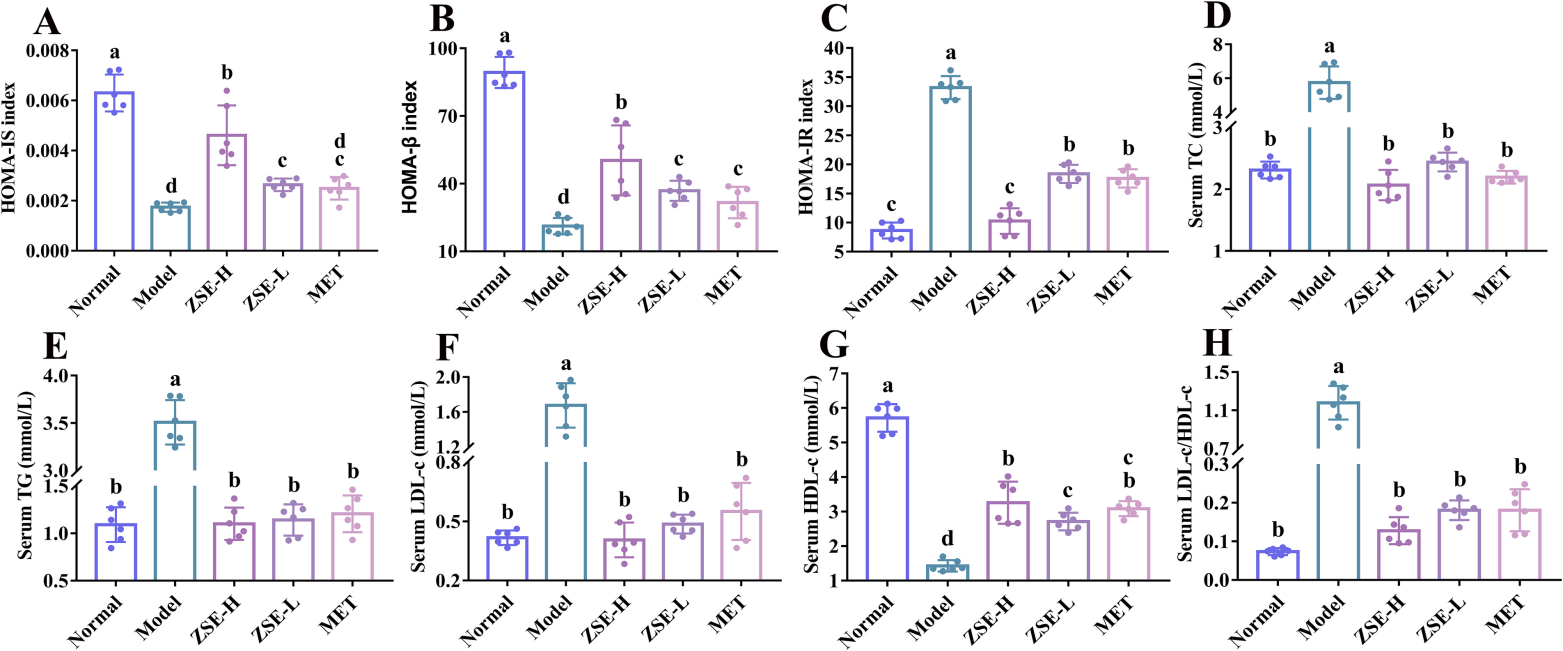
Pancreatic islet function correlation indices and serum biochemical indicators. **(A)** HOMA-IS, **(B)** HOMA-β, **(C)** HOMA-IR, **(D)** serum TC, **(E)** serum TG, **(F)** serum LDL-c, **(G)** serum HDL-c, and **(H)** serum LDL-c/HDL-c.

Homeostasis model assessment (HOMA) is a widely used method for evaluating pancreatic islet function, including the quantitative analysis of β-cell function, assessment of insulin resistance, and detection of insulin sensitivity. The HOMA-IS and HOMA-β index in the ZSE-H and ZSE-L groups were significantly higher than those in the model group (*p* < 0.05), with the ZSE-H group showing significantly higher level than both the ZSE-L and MET groups (*p* < 0.05) (Figures 2A,B). Additionally, no significant difference was observed between the MET and ZSE-L groups (*p* > 0.05). As shown in Figure 2C, the HOMA-IR index in all treatment groups were significantly lower than those in the model group (*p* < 0.05). The ZSE-H group exhibited significantly lower HOMA-IR index compared to the ZSE-L and MET groups (*p* < 0.05). In addition to hyperglycemia, T2DM can cause lipid metabolism disorders, leading to various biochemical abnormalities. As illustrated in Figures 2D–F, the levels of serum TC, TG, and LDL-c were significantly reduced in the treatment groups compared to the model group (*p* < 0.05). Conversely, the level of serum HDL-c was significantly increased in the normal, ZSE-H, ZSE-L, and MET groups compared to the model group (*p* < 0.05) (Figure 2G). Notably, the serum HDL-c level in the ZSE-H group was significantly higher than in the ZSE-L group (*p* < 0.05). Since single biochemical indicator may be insufficient to assess T2DM risk, the LDL-c/HDL-c ratio was used as a key predictor for evaluating lipid metabolism disorders. The LDL-c/HDL-c ratio in the normal, ZSE-H, ZSE-L, and MET groups was significantly lower compared to the model group (*p* < 0.05) (Figure 2H).

### 3.4 Effects of ZSE on liver biochemical indicators and histopathological characteristics

**Figure 3:**
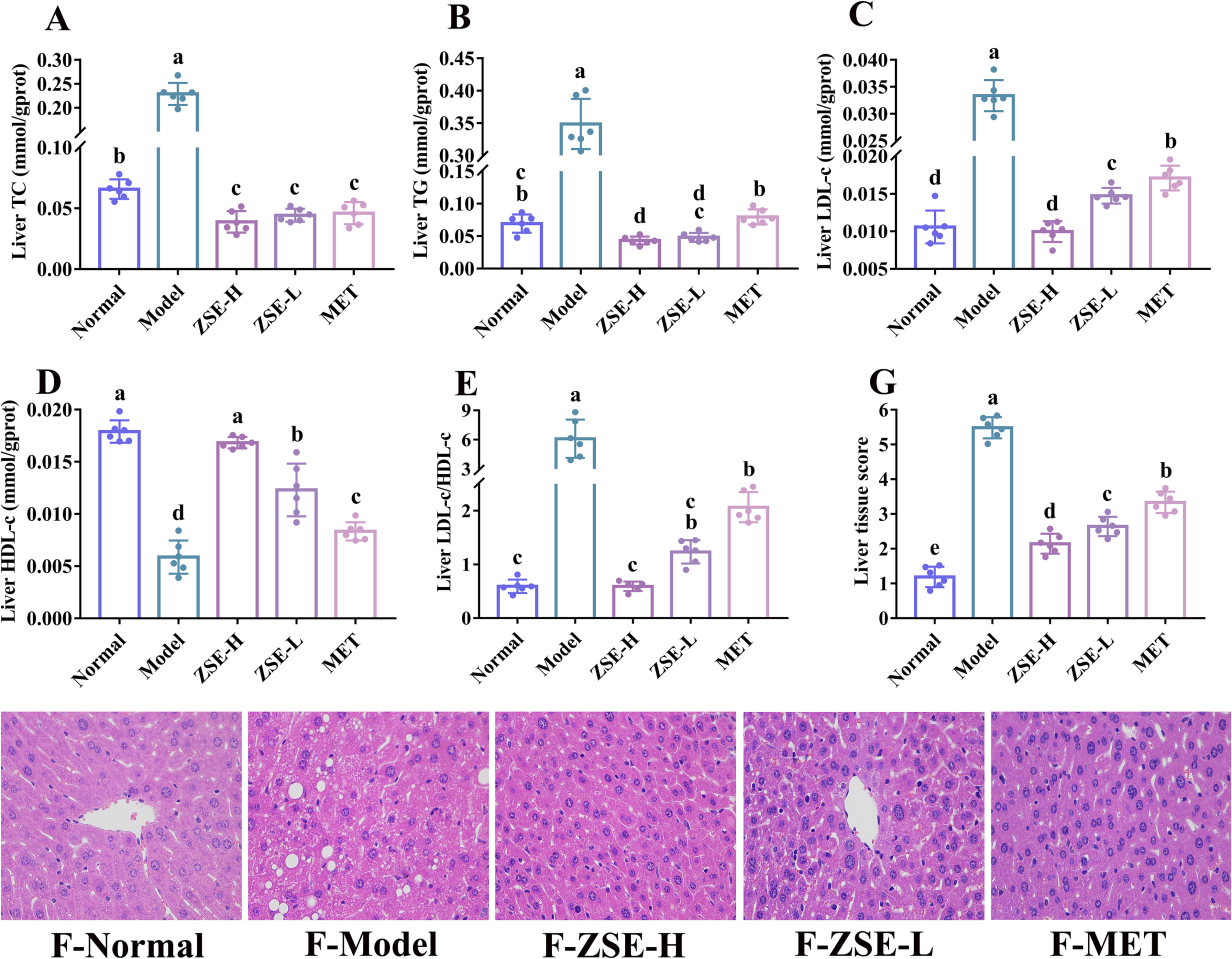
Biochemical indicators and histopathological analysis of liver tissue. **(A)** Liver TC, **(B)** liver TG, **(C)** liver LDL-c, **(D)** liver HDL-c, **(E)** liver LDL-c/HDL-c, **(F)** liver histopathology (magnification 400×), and **(G)** liver tissue score.

Lipid metabolism disorders are closely associated with liver injury, as the liver plays a central role in lipid metabolism, producing various lipids such as TC, TG, LDL-c, and HDL-c. Disruptions in lipid metabolism can lead to the excessive accumulation of liver fat, tissue inflammation, and other adverse effects. The liver TC and TG levels were significantly reduced in the treatment groups compared to the model group (*p* < 0.05) (Figures 3A,B). Furthermore, the liver TG level in the ZSE-H and ZSE-L groups were notably lower than in the MET group (*p* < 0.05), with the ZSE-H group showing a significantly lower level than the normal group (*p* < 0.05) (Figure 3B). Liver LDL-c level in the treatment groups were significantly reduced compared to the model group (*p* < 0.05), with the ZSE-H group showing a significantly lower level than the ZSE-L group (*p* < 0.05) (Figure 3C). Conversely, Figure 3D shows a significant increase in liver HDL-c level in the treatment groups compared to the model group (*p* < 0.05), and the ZSE-H group exhibited significantly higher HDL-c level than the ZSE-L group (*p* < 0.05). The LDL-c/HDL-c ratio was also employed to further evaluate the extent of lipid metabolism disorders in the liver. In Figure 3E, the LDL-c/HDL-c ratio in the treatment groups was significantly lower than that in the model group (*p* < 0.05), with the ZSE-H group displaying a significantly lower ratio than the MET group (*p* < 0.05).

Histomorphological analysis provides further insight into liver injury, examining features such as cell morphology, tissue organization, and lipid accumulation (Figure 3F). In the normal group, the hepatic cord structure exhibited a clear and orderly radial arrangement, with well-defined, intact nuclei and no pathological changes. In contrast, the model group showed disordered hepatic cords, characteristic of nonalcoholic fatty liver disease, with hepatocytes filled with large lipid vacuoles, leading to fat accumulation and further structural damage. Hepatocyte nuclei were irregular in size, exacerbating cellular injury. After 4 weeks of ZSE-H treatment, significant improvements were observed in the liver morphology of T2DM mice. The number of lipid vacuoles was markedly reduced, hepatic nuclei were uniform in size, cell arrangement was neat, and hepatic cord structure was restored. While the ZSE-L group also showed a reduction in lipid vacuoles, inconsistencies in nuclear size remained. In the MET group, some lipid vacuoles persisted, and nuclear irregularities were still evident. Liver tissue scoring is a crucial method for evaluating the severity of liver disease and pathological changes. This assessment includes factors such as hepatocyte steatosis, inflammatory lesions in hepatic lobules, hepatocyte injury, liver fibrosis, and lipid droplet vacuole presence. Each category is scored from 0 to 3 based on severity, with higher scores reflecting more severe symptoms. A comprehensive tissue score was calculated by summing the individual scores to fully represent the pathological status of the liver. As shown in Figure 3G, liver tissue score in the ZSE-H, ZSE-L, and MET groups were significantly lower than in the model group (*p* < 0.05) but higher than in the normal group (*p* < 0.05). Additionally, the ZSE-H and ZSE-L groups had significantly lower scores than the MET group (*p* < 0.05), with the ZSE-H group showing significantly lower scores than the ZSE-L group (*p* < 0.05).

### 3.5 qPCR and immunohistochemical analysis

**Figure 4:**
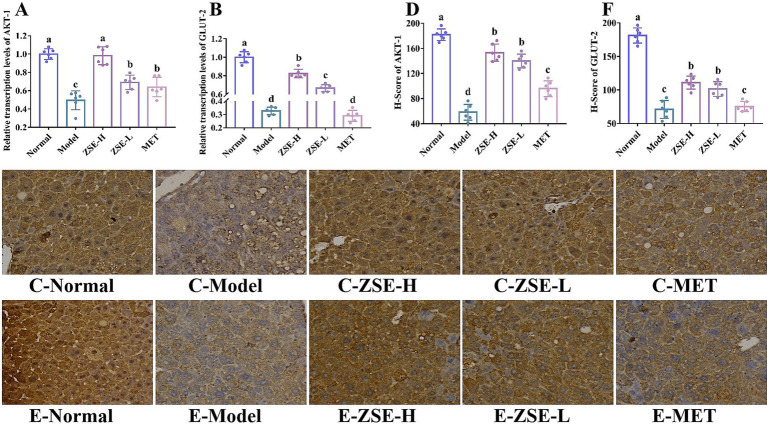
Effects of ZSE on mRNA transcription levels. **(A)** AKT-1 and **(B)** GLUT-2. Immunohistochemical analysis and H-score of **(C,D)** AKT-1 and **(E,F)** GLUT-2.

The objective of this study was to evaluate the impact of ZSE on the relative transcription levels of AKT-1 and GLUT-2 in liver tissue. As depicted in Figures 4A,B, the relative transcription levels of AKT-1 and GLUT-2 were significantly elevated in the ZSE-H and ZSE-L groups compared to the model group (*p* < 0.05), with the ZSE-H group showing a markedly higher increase than the ZSE-L and MET groups (*p* < 0.05). Immunohistochemical (IHC) analysis, a widely used technique in pathological diagnostics, relies on the highly specific interaction between antibodies and antigens to quantify and analyze antigen expression in liver tissue sections. Positive cell counts and staining intensity in IHC sections were quantified using histochemistry scores (H-scores) to achieve both qualitative and relative quantitative assessments. IHC was employed to investigate the expression of AKT-1 and GLUT-2 in liver tissue. As shown in Figures 4C–E, after 4 weeks of ZSE treatment, both AKT-1 and GLUT-2 were expressed with a brownish-yellow hue, localized within the cytoplasm or nucleus. In contrast, the expression of these markers was reduced in the model group. H-score analysis revealed that the H-score of AKT-1 and GLUT-2 in the ZSE-H and ZSE-L groups were significantly higher than in the model and MET groups (*p* < 0.05) (Figures 4D–F).

### 3.6 Effects of ZSE on intestinal microbiota and SCFAs in cecum contents of mice

**Figure 5:**
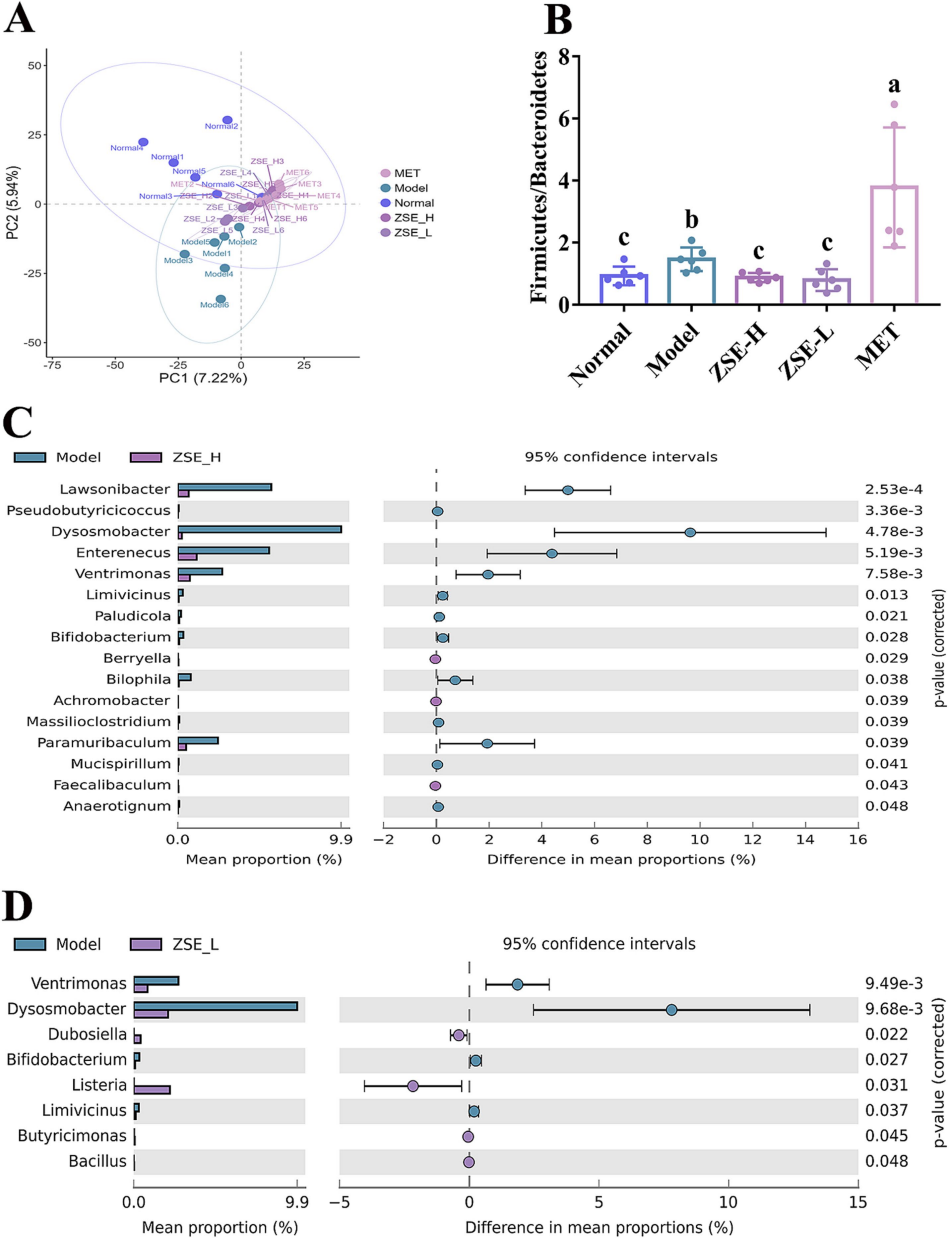
Effects of ZSE treatment on intestinal microbial communities in the cecum. **(A)** PCA plot, **(B)** Firmicutes/Bacteroidetes ratio. Extended error bar plot showing significant differences in intestinal microbiota at the genus level: **(C)** ZSE-H vs. model; **(D)** ZSE-L vs. model.

The distinct separation of sample distribution is observed between the normal and model groups, with normal group samples clustering in the first and second quadrants, far from the model group (Figure 5A). After 4 weeks of ZSE intervention, the ZSE-H and ZSE-L groups exhibited intergroup distributions that were also distinct from the model group. Firmicutes and Bacteroidetes are the dominant bacterial phyla in the gut, and Firmicutes/Bacteroidetes ratio is closely related to hyperglycemia in T2DM. The Firmicutes/Bacteroidetes ratios across groups showed a significant reduction in this ratio in the normal, ZSE-H, and ZSE-L groups compared to the model group (*p* < 0.05) (Figure 5B). Notably, the Firmicutes/Bacteroidetes ratio in the MET group was significantly higher than in the other groups (*p* < 0.05). At the genus level, differences in the relative abundance of bacterial genera between the ZSE-H, ZSE-L, and model groups were analyzed to assess changes in gut microbiota following 4 weeks of ZSE treatment in T2DM mice. Figure 5C highlights bacterial genera that exhibited significant differences between the ZSE-H and model groups. Compared to the model group, the relative abundance of *Berryella*, *Achromobacter*, and *Faecalibaculum* was significantly increased in the ZSE-H group (*p* < 0.05), while the relative abundance of *Lawsonibacter*, *Pseudobutyricicoccus*, *Dysosmobacter*, *Enterenecus*, *Ventrimonas*, *Limivicinus*, *Paludicola*, *Bifidobacterium*, *Bilophila*, *Massilioclostridium*, *Paramuribaculum*, *Mucispirillum*, and *Anaerotignum* was significantly decreased (*p* < 0.05). In Figure 5D, the bacterial genera with significant differences between the ZSE-L and model groups are depicted. In the ZSE-L group, the relative abundance of *Dubosiella*, *Listeria*, *Butyricimonas*, and *Bacillus* was significantly increased compared to the model group (*p* < 0.05), while the relative abundance of *Ventrimonas*, *Dysosmobacter*, *Bifidobacterium*, and *Limivicinus* was significantly reduced (*p* < 0.05).

**Figure 6:**
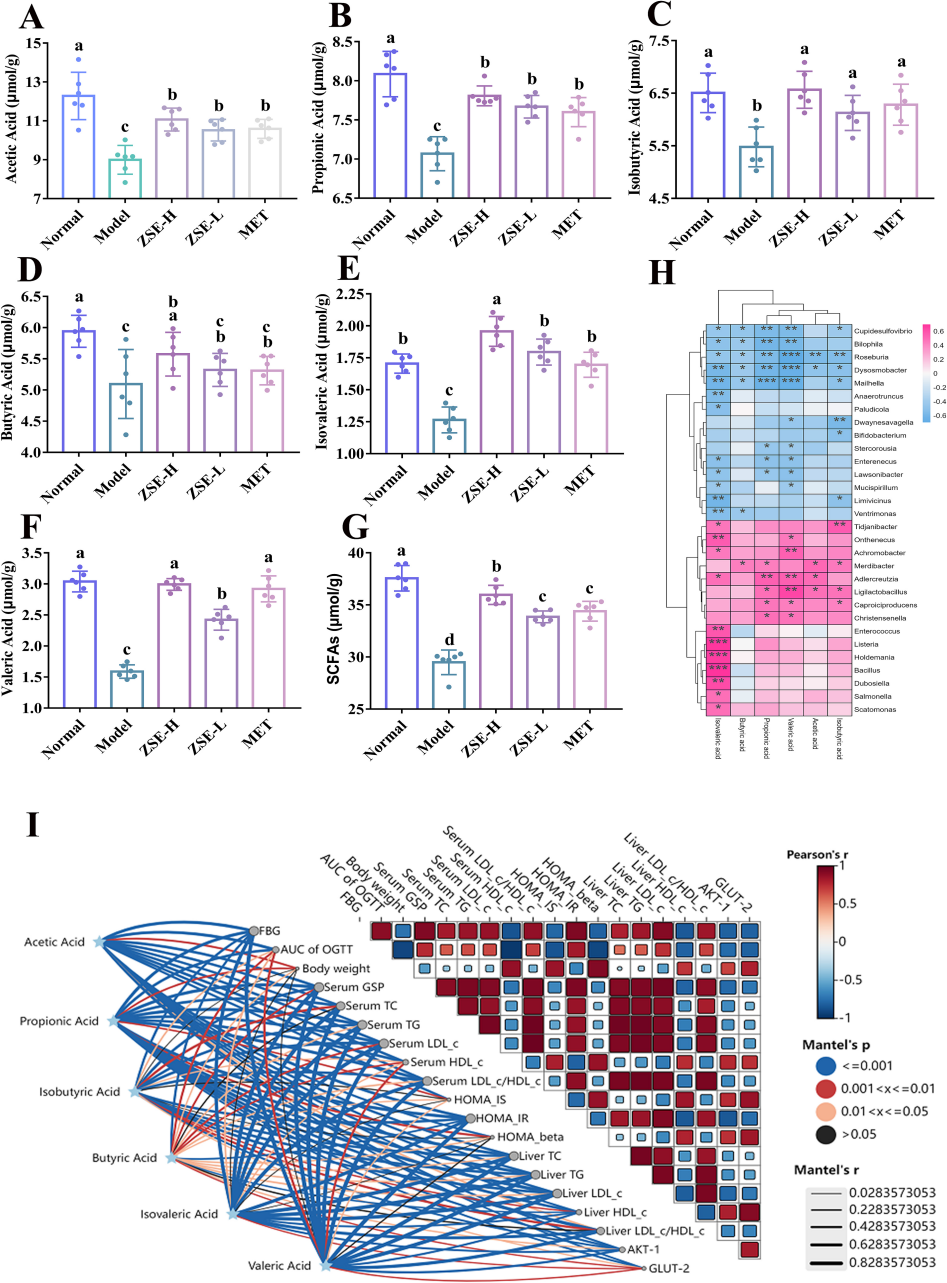
Effects of ZSE treatment on SCFAs content in the cecum. **(A)** Acetic acid, **(B)** propionic acid, **(C)** isobutyric acid, **(D)** butyric acid, **(E)** isovaleric acid, **(F)** valeric acid, **(G)** total SCFAs. **(H)** Spearman correlation heat map between bacterial genera and SCFAs. **(I)** Mantel test analysis showing the correlation between hypoglycemic parameters and SCFAs.

SCFAs, including acetic acid, propionic acid, isobutyric acid, butyric acid, isovaleric acid, and valeric acid, are key metabolites produced by the intestinal microbiota through carbohydrate fermentation. These metabolites play critical roles in maintaining intestinal barrier integrity, regulating glucose and lipid metabolism, and mitigating systemic inflammation. Figure 6 illustrates the specific changes in SCFAs content in the cecum of T2DM mice following 4 weeks of ZSE treatment. As shown in Figures 6A,B, the levels of acetic acid and propionic acid in the ZSE-H, ZSE-L, and MET groups were significantly higher than in the model group (*p* < 0.05). Figure 6C indicates that the isobutyric acid content was significantly elevated in all treatment groups compared to the model group (*p* < 0.05). In Figure 6D, butyric acid level in the normal and ZSE-H groups were significantly higher than in the model group (*p* < 0.05). The levels of isovaleric and valeric acid were significantly increased in the ZSE-H, ZSE-L, and MET groups compared to the model group (*p* < 0.05) (Figures 6E,F), with the ZSE-H group exhibiting significantly higher levels than the ZSE-L group (*p* < 0.05). The total SCFAs content across groups is depicted in Figure 6G, where the total SCFAs in the ZSE-H, ZSE-L, and MET groups were significantly higher than in the model group (*p* < 0.05). Moreover, the ZSE-H group displayed significantly higher SCFAs level than both the ZSE-L and MET groups (*p* < 0.05).

To further investigate the relationship between SCFAs and gut microbiota at the genus level, a correlation heatmap was generated (Figure 6H). *Ligilactobacillus* exhibited a significant positive correlation with acetic acid, propionic acid, isobutyric acid, and valeric acid (*p* < 0.05), while *Christensenella* showed a significant positive correlation with propionic and valeric acid (*p* < 0.05). In contrast, *Mucispirillum* was negatively correlated with valeric and isovaleric acid (*p* < 0.05), and *Anaerotruncus* displayed a significant negative correlation with isovaleric acid (*p* < 0.01). The Mantel test analysis (Figure 6I) further revealed significant associations between SCFAs and various hypoglycemic parameters, including FBG, AUC of OGTT, serum GSP, serum TG, serum LDL-c, serum HDL-c, serum LDL-c/HDL-c, HOMA-IR, liver TC, liver TG, liver LDL-c, liver HDL-c, as well as AKT-1 and GLUT-2 (*p* < 0.05).

**Figure 7:**
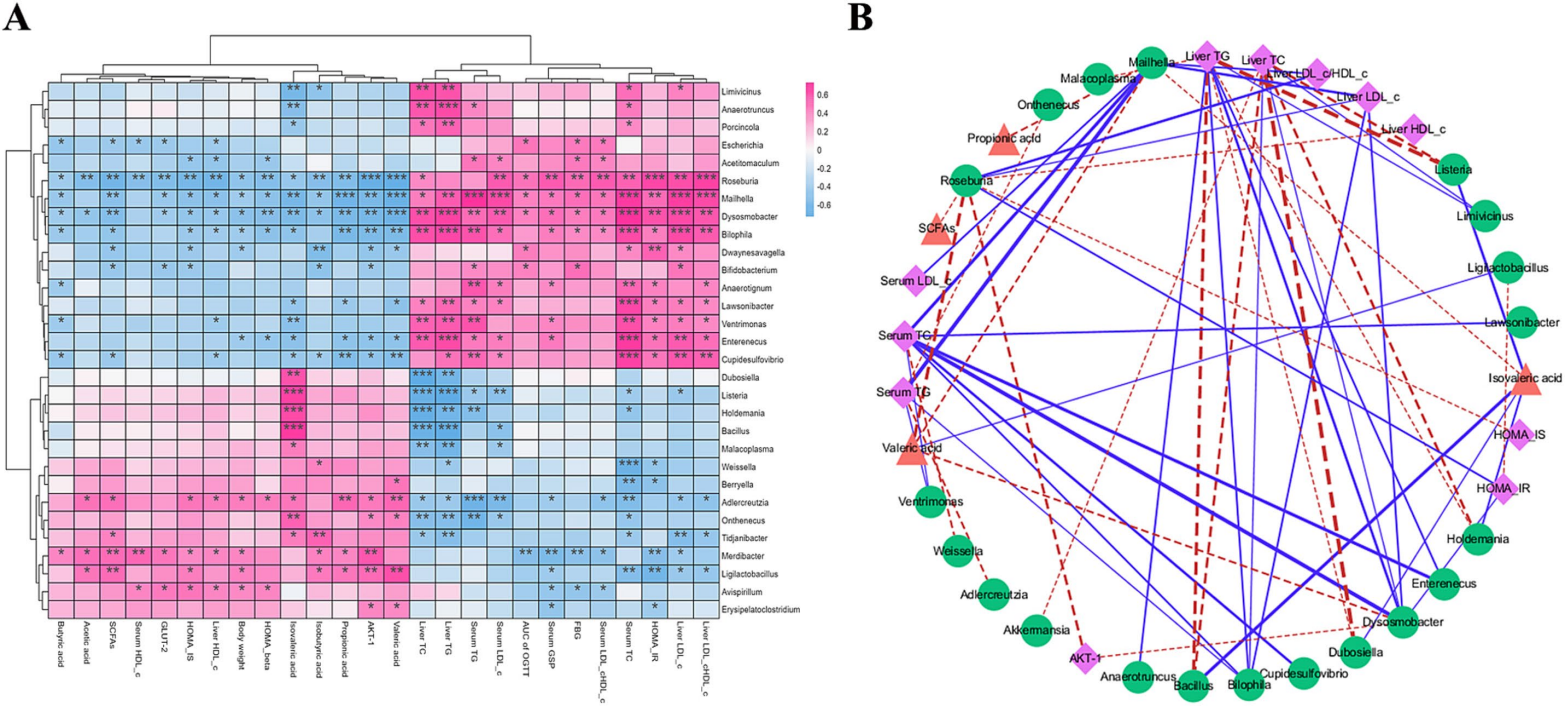
Hierarchical clustering analysis **(A)** and correlation network visualization **(B)** using Spearman correlation between hypoglycemic parameters and representative bacterial genera. In **(A)**, deep pink indicates positive correlations, while light sky blue indicates negative correlations, with color depth reflecting the strength of correlation. In **(B)**, pink, green, and orange-red indicate hypoglycemic parameters, bacterial genera, and SCFAs, respectively. Blue solid lines represent *r* > 0.6 with adjusted *p* < 0.01, and red dotted lines represent *r* < −0.6 with adjusted *p* < 0.01.

This study explored the relationship between specific representative differential bacterial genera and hypoglycemic parameters following 4 weeks of ZSE intervention (Figure 7A). *Ligilactobacillus* demonstrated a significant positive correlation with acetic acid, SCFAs, HOMA-IS, body weight, isobutyric acid, propionic acid, AKT-1, and valeric acid (*p* < 0.05), while exhibiting a significant negative correlation with serum GSP, serum TC, HOMA-IR, liver LDL-c, and the liver LDL-c/HDL-c (*p* < 0.05). *Anaerotruncus* displayed a significant positive correlation with TC and TG levels in both serum and liver (*p* < 0.05), and a negative correlation with isovaleric acid (*p* < 0.01). The use of Cytoscape provided deeper insights into the intrinsic relationships between hyperglycemia status and intestinal microbial communities. As illustrated in Figure 7B, parameters with a correlation coefficient |*r*| ≥ 0.6 were selected for further analysis. *Ligilactobacillus* showed a strong negative correlation with HOMA-IR (*r* = −0.621) and a strong positive correlation with valeric acid (*r* = 0.62). *Anaerotruncus* exhibited a strong positive correlation with liver TG (*r* = 0.634). Furthermore, *Dubosiella*, *Bacillus*, and *Mailhella* exhibited significant correlations with multiple hypoglycemic parameters. For example, *Dubosiella* and *Bacillus* were strongly negatively correlated with liver TC (*r* = −0.721 and *r* = −0.662, respectively) and liver TG (*r* = −0.619 and *r* = −0.706, respectively), while both showed a strong positive correlation with isovaleric acid (*r* = 0.604 and *r* = 0.696, respectively). In contrast, *Mailhella* displayed a strong positive correlation with serum TC, serum TG, serum LDL-c, liver LDL-c, and the liver LDL-c/HDL-c ratio (*r* = 0.694, 0.734, 0.634, 0.687, and 0.643, respectively), and a strong negative correlation with propionic acid, isovaleric acid, and valeric acid (*r* = −0.635, −0.606, and −0.653, respectively).

## 4 Discussion

T2DM, a global endocrine and metabolic disorder, poses an increasing and significant threat to human health (24). In China, the incidence of T2DM has surged due to the accelerated aging of the population. Moreover, T2DM is often accompanied by a range of acute and chronic complications, such as diabetic ketoacidosis, atherosclerosis, and hepatitis (25). In recent years, the rapid development of society and improvements in living standards have led to the widespread adoption of HSHF diets, which are key contributors to T2DM and its associated complications. This unhealthy dietary pattern can result in elevated FBG levels and pancreatic islet dysfunction, both of which are hallmark indicators of pre-diabetes. More critically, persistent insulin resistance in T2DM keeps FBG and 2-h postprandial blood glucose levels consistently high, directly impacting liver function. The consumption of an HSHF diet promotes abnormal fat deposition in the liver, triggering inflammation and structural injury to liver tissue. Concurrently, liver injury reduces the sensitivity of insulin receptors, exacerbating insulin resistance and further impairing liver function. This vicious cycle not only disrupts the liver’s normal physiological functions but also accelerates the progression of T2DM (26). Therefore, lifestyle interventions, such as a healthy diet and regular exercise, are essential for preventing and managing T2DM and its complications.

When blood glucose level rise abnormally, the body’s ability to efficiently absorb and metabolize glucose and other essential energy substrates is impaired, preventing these substances from being utilized to maintain physiological functions. Instead, they are excreted through urine or feces (27). This process ultimately leads to abnormal weight loss in T2DM mice. In this study, prior to ZSE treatment (week 0), the body weight of T2DM mice was significantly lower than that of the normal group, indicating a reduced capacity for energy uptake and utilization in T2DM mice. After 4 weeks of ZSE intervention, the body weight of both the ZSE-H and ZSE-L groups showed a significant recovery. These results suggest that ZSE-H and ZSE-L can enhance the absorption and utilization of glucose and other energy substrates, thereby reducing their loss through excretion and alleviating abnormal weight loss symptoms in T2DM mice. Notably, no significant difference in body weight was observed between the MET and model groups at weeks 2 and 4 of MET intervention. This phenomenon may be attributed to MET-induced stimulation of intestinal 5-hydroxytryptamine production, which acts on the central nervous system to cause diarrhea in mice, thereby inhibiting the normal absorption and utilization of energy substrates in the intestine. As a result, the body weight of the MET group did not show a significant recovery (28).

Monitoring body weight is critical for understanding the growth patterns and potential disease progression in mice, while FBG level serve as an important metric for assessing T2DM status. After 2 and 4 weeks of ZSE intervention, FBG level in T2DM mice were significantly reduced, with the ZSE-H group showing a greater reduction than the ZSE-L and MET groups. This suggests that both ZSE-H and ZSE-L possess hypoglycemic properties, with ZSE-H exhibiting a more pronounced effect. Although FBG is a common clinical indicator for the preliminary screening and diagnosis of T2DM, it has certain limitations. Specifically, it reflects blood glucose levels only at the time of measurement, and physiological fluctuations may affect its accuracy. As such, relying on an FBG level ≥7.0 mmol/L as the diagnostic threshold for T2DM, particularly when readings are near this threshold, can increase the risk of misdiagnosis or missed diagnosis (9). To address these limitations and enhance diagnostic accuracy, researchers have explored more comprehensive detection strategies. Jiao et al. (29) proposed an integrated framework that includes key indicators such as OGTT, AUC of OGTT, and serum GSP, providing a more robust foundation for the early diagnosis and treatment of T2DM. OGTT assesses the body’s ability to regulate blood glucose level following glucose intake, evaluating glucose tolerance and insulin secretion. Its diagnostic criteria focus on deviations in blood glucose level at specific time points, while the AUC of OGTT offers a more thorough and precise evaluation by considering blood glucose levels at four different time points. This comprehensive approach improves the reliability of OGTT results. Additionally, serum GSP remains unaffected by transient fluctuations in blood glucose, offering a stable and accurate reflection of glucose management over the preceding 2–3 weeks, which further aids in the diagnosis of T2DM (30). In this study, the OGTT curves for the ZSE-H and ZSE-L groups fell between those of the normal and model groups. Moreover, significant reductions in AUC of OGTT and serum GSP were observed in T2DM mice treated with ZSE-H and ZSE-L over 4 weeks. Based on the data from body weight, FBG, OGTT, AUC of OGTT, and serum GSP, ZSE demonstrated a marked improvement in hyperglycemia symptoms in T2DM mice, with ZSE-H exhibiting superior efficacy compared to ZSE-L in terms of FBG and AUC of OGTT.

The medical community now widely recognizes that the primary cause of T2DM is the impaired function of pancreatic β cells, and the extent of this dysfunction is closely associated with the progression of the disease (31). Moreover, a prolonged decline in pancreatic β-cell function can induce and exacerbate insulin resistance (32). Therefore, evaluating pancreatic islet function through indicators such as HOMA-IS, HOMA-IR, and HOMA-β via the HOMA method is crucial for the early prevention, monitoring, and development of effective treatment strategies for T2DM. The HOMA model is a key method for assessing pancreatic islet function by referencing FBG and serum insulin levels to calculate HOMA-IS, HOMA-β, and HOMA-IR, thereby offering a systematic and comprehensive evaluation of insulin sensitivity, β-cell function, and insulin resistance (33). This method is widely applied in clinical practice and provides a robust scientific foundation for diagnosing T2DM, monitoring treatment efficacy, and formulating preventive strategies.

In this study, following 4 weeks of ZSE intervention, significant improvements were observed in HOMA-IS, HOMA-IR, and HOMA-β, with the ZSE-H group demonstrating superior results compared to the ZSE-L group. These results suggest that ZSE at varying doses can significantly restore pancreatic islet function in T2DM mice, with the high-dose ZSE-H group exhibiting the most pronounced effect. Additionally, impaired islet function leads to an increase in endogenous triglyceride synthesis in the liver. This occurs as glucose uptake and utilization by peripheral tissues decline, promoting lipolysis and transporting more free fatty acids to the liver. These free fatty acids are subsequently converted into triglycerides and stored (34). This process exacerbates lipid accumulation in the liver and further deteriorates lipid metabolism parameters.

In the research, T2DM mice exhibited clear signs of lipid metabolism disorder, accompanied by pathological features of liver tissue injury, supporting the notion that T2DM contributes to abnormal lipid metabolism and liver damage. Specifically, TC, TG, and LDL-c levels in the serum and liver of T2DM mice were significantly elevated, while HDL-c levels were markedly reduced, indicating severe disruption of lipid metabolism. Additionally, research by Zou et al. (35) highlighted LDL-c/HDL-c as an independent predictor of metabolic disorders such as nonalcoholic fatty liver disease and T2DM, with predictive power surpassing that of either HDL-c or LDL-c alone. This provides a more accurate risk assessment tool for clinical practice. In this research, ZSE significantly improved TC, TG, LDL-c, HDL-c, and LDL-c/HDL-c levels in both serum and liver of T2DM mice, indicating that ZSE has a beneficial effect on lipid metabolism disorders associated with T2DM. Furthermore, ZSE-H demonstrated superior effects compared to ZSE-L in enhancing serum HDL-c, as well as liver LDL-c and HDL-c levels. Histopathological analysis of the liver revealed that the hepatic cord structure in T2DM mice was disordered, likely due to factors such as hepatocyte injury, edema, or inflammatory response. Additionally, hepatocytes exhibited significant lipid droplet accumulation, indicative of substantial fat deposition. This accumulation not only increases the burden on the liver but also raises the risk of liver diseases, such as nonalcoholic fatty liver disease, further confirming the close association between T2DM-induced lipid metabolism disorders and liver tissue injury (36). The histopathological findings suggest that ZSE ameliorates liver injury caused by lipid metabolism dysfunction. The activation of AKT-1 leads to an upregulation of GLUT-2 expression in liver tissue, enhancing the liver’s capacity to absorb glucose from the bloodstream. This, in turn, promotes intracellular energy metabolism and increases the rate of glucose consumption, effectively reducing blood glucose levels. This mechanism is instrumental in alleviating hyperglycemia in patients with T2DM (37). Therefore, assessing the relative expression of AKT-1 and GLUT-2 in liver tissue is essential for understanding how ZSE improves T2DM symptoms and for developing related therapeutic strategies. In this study, combined with FBG, OGTT, liver histopathology, and other indicators, the results indicated that ZSE significantly elevated the relative transcription levels of AKT-1 and GLUT-2, as well as the H-score in the liver tissue of T2DM mice. These results suggest that ZSE enhances the liver’s capacity to absorb glucose from the bloodstream, thereby improving hyperglycemia symptoms in T2DM mice.

The structural homeostasis of intestinal microbiota is closely linked to the host’s physiological functions, particularly in maintaining normal glucose metabolic pathways. Significant differences in intestinal microbial communities have been observed between healthy individuals and patients with T2DM, such as a marked increase in the ratio of Firmicutes/Bacteroides in patients with T2DM. Principal component analysis (PCA), an effective statistical method, can visually depict the variation trends within a group and the feature differences across groups, thereby revealing intra-group and inter-group differences. In this study, PCA results indicated that, following 4 weeks of ZSE treatment, the microbiota samples were clearly separated from those in the model group, suggesting a regulatory effect of ZSE on the intestinal microbial communities of T2DM mice. At the phylum level, the Firmicutes/Bacteroidetes ratio in T2DM mice decreased significantly after 4 weeks of ZSE treatment. This finding demonstrates that both ZSE-H and ZSE-L effectively restored the intestinal microbial communities at the phylum classification level, aligning with the results reported by Yang et al. (38). Notably, the Firmicutes/Bacteroidetes ratio in the MET group was significantly higher than in other groups, potentially due to MET-induced intestinal barrier damage, which disrupts intestinal barrier function and disturbs microbial communities. Similar findings have been reported by Petakh et al. (39). At the genus level, the classification and function of microbiota become more complex and diverse. Zhu et al. (40) found that myricetin intervention in diabetic cardiomyopathy mice significantly increased the relative abundance of *Faecalibaculum* in the gut, a result consistent with the present study, where ZSE-H significantly increased *Faecalibaculum* abundance in T2DM mice. Additionally, ZSE-H significantly decreased the relative abundance of *Bilophila* in the intestinal tract of T2DM mice. This is consistent with findings by Ke et al. (41), who demonstrated that sulfated galactofucan from *Undaria pinnatifida* reduced the relative abundance of *Bilophila* in T2DM mice, alleviating hyperglycemia symptoms. Furthermore, Li et al. (42) explored the regulatory effects of mulberry leaf Fu tea on metabolic disorders and intestinal microbial communities in T2DM mice, finding that the tea significantly ameliorated disturbances in microbial communities and increased the relative abundance of beneficial bacteria such as *Dubosiella*. This is in line with our research, where ZSE-L significantly increased the relative abundance of *Dubosiella* in T2DM mice.

SCFAs are metabolites produced by intestinal microbiota through the fermentation of non-digestible carbohydrates, with chain lengths ranging from 1 to 6 carbon atoms. These include acetic acid, propionic acid, isobutyric acid, butyric acid, isovaleric acid, and valeric acid. SCFAs are absorbed by intestinal epithelial cells, providing energy for various tissues (43). Studies have shown that increased SCFAs levels have a positive impact on intestinal health by promoting the proliferation of probiotics and reducing the relative abundance of harmful bacteria, thereby maintaining intestinal microecological balance (11). Acetic acid, the most abundant SCFA in the gut, not only participates in lipid and cholesterol biosynthesis but also mitigates lipid deposition in the liver and enhances its metabolic efficiency (14). Propionic acid, primarily absorbed by the liver, improves the sensitivity of pancreatic β cells to glucose by inhibiting cell apoptosis, thereby supporting normal pancreatic function (42). Butyric acid helps regulate blood glucose levels by enhancing glucose uptake in muscle and fat tissues (44). Hence, SCFAs play a critical role in maintaining health by acting on the intestine and other tissues through various pathways.

In this study, after 4 weeks of ZSE-H treatment, the levels and total content of SCFAs in T2DM mice were significantly increased, with isovaleric acid, valeric acid, and total SCFAs levels in the ZSE-H group being markedly higher than those in the ZSE-L group. These results indicate that ZSE-H significantly elevates SCFAs content in the cecum of T2DM mice. Additionally, at the genus level, correlation heat maps revealed a relationship between microbiota and SCFAs. Hu et al. (45) reported that mulberry leaf oligosaccharides selectively promote the proliferation of symbiotic bacteria such as *Ligilactobacillus*, thereby alleviating hyperglycemia in T2DM. Furthermore, Yuan et al. (46) found a positive correlation between *Ligilactobacillus* and SCFAs, including acetic acid, propionic acid, isobutyric acid, butyric acid, and valeric acid, consistent with the significant positive correlation between *Ligilactobacillus* and these SCFAs in this study. Cuffaro et al. (47) demonstrated that *Christensenella* plays a pivotal role in treating obesity and T2DM, while Borrelli et al. (48) reported a positive correlation between *Christensenella* and propionic and butyric acids. These findings align with the significant positive correlation observed between *Christensenella* and propionic acid in this research. Moreover, Li et al. (42) found that SCFAs were negatively correlated with indicators such as AUC of OGTT and HOMA-IR in T2DM mice treated with mulberry leaf Fu tea, consistent with the correlations between SCFAs and hypoglycemic parameters observed in this study.

To further investigate the effects of ZSE on T2DM-induced hyperglycemia through the regulation of intestinal microbial communities in mice, hierarchical clustering was employed to systematically analyze the correlation between bacteria and hypoglycemic parameters. Crusell et al. (49) identified a positive correlation between *Anaerotruncus* and FBG, along with a negative correlation between *Anaerotruncus* and HOMA-IS. Huang et al. (50) explored the association between intestinal microbiota and SCFAs, finding that *Dubosiella* exhibited a negative correlation with valeric acid and isovaleric acid. Yan et al. (51) examined the impact of *Bacillus* on the intestinal microbiota of T2DM mice and found that it not only reduced FBG level but also alleviated insulin resistance and improved lipid metabolism disorders. Similarly, Xiao et al. (52) reported that *Termite fungus comb* polysaccharides reduced the relative abundance of *Mailhella*, thereby mitigating glycolipid metabolism disorders and exerting an anti-diabetic effect. In this study, *Dubosiella*, *Bacillus*, and *Mailhella* were found to be strongly correlated with multiple hypoglycemic parameters, suggesting their potential as intestinal marker bacteria for further investigation into the development and progression of T2DM. The findings indicate that both ZSE-H and ZSE-L effectively alleviated hyperglycemia symptoms in T2DM mice. Across multiple indicators, including FBG, AUC of OGTT, HOMA-IS, HOMA-IR, HOMA-β, serum HDL-c, liver LDL-c, liver HDL-c, relative transcription levels of AKT-1 and GLUT-2, as well as isovaleric acid and valeric acid, the improvement observed in the ZSE-H group was significantly greater than in the ZSE-L group. Furthermore, this study confirmed that ZSE plays a pivotal role in ameliorating hyperglycemia symptoms in T2DM mice by modulating the composition of intestinal microbial communities.

## 5 Conclusion

This study, utilizing a T2DM mouse model, demonstrated that a 4-week intervention with ZSE significantly alleviated liver injury and improved body weight, FBG, AUC of OGTT, serum GSP, HOMA-IS, HOMA-IR, and HOMA-β levels in T2DM mice. Notably, the improvements in FBG, AUC of OGTT, HOMA-IS, HOMA-IR, HOMA-β, serum HDL-c, liver LDL-c, liver HDL-c, isovaleric acid, valeric acid, and the relative transcription levels of AKT-1 and GLUT-2 were markedly greater in the ZSE-H group compared to the ZSE-L group. Furthermore, at the phylum level, both ZSE-H and ZSE-L groups exhibited a significant reduction in the Firmicutes/Bacteroidetes ratio, while at the genus level, ZSE-H increased the relative abundance of beneficial bacteria such as *Faecalibaculum* and reduced the relative abundance of harmful bacteria like *Bilophila*. Additionally, strong correlations were observed between *Dubosiella*, *Bacillus*, *Mailhella*, and key hypoglycemic parameters, suggesting their potential as intestinal marker bacteria in T2DM progression. In conclusion, these findings provide a robust scientific foundation for further exploration of ZSE’s potential in alleviating hyperglycemia symptoms in patients with T2DM.

## Data Availability

The data presented in the study is deposited in NCBI, accession number is PRJNA1200150: https://www.ncbi.nlm.nih.gov/bioproject/PRJNA1200150/.
